# Neuroinflammation and brain–peripheral interaction in ischemic stroke: A narrative review

**DOI:** 10.3389/fimmu.2022.1080737

**Published:** 2023-01-05

**Authors:** Wenjing Cheng, Qing Zhao, Chengzhen Li, Yunzhi Xu

**Affiliations:** ^1^ Department of Laboratory Medicine, Linping Hospital of Integrated Traditional Chinese and Western Medicine, Hangzhou, Zhejiang, China; ^2^ Center for Translational Medicine, Shanghai Jiao Tong University Affiliated Sixth People’s Hospital, Shanghai, China; ^3^ Department of Laboratory Medicine, Shanghai Guanghua Hospital of Integrated Traditional Chinese and Western Medicine, Guanghua Hospital Affiliated to Shanghai University of Traditional Chinese Medicine, Shanghai, China; ^4^ Department of Laboratory Medicine, Wenzhou Central Hospital, Affiliated Dingli Clinical Institute of Wenzhou Medical University, Wenzhou, China

**Keywords:** ischemic stroke, immunity, inflammation, comorbidity, immunotherapy, review

## Abstract

Excessive immune activation within the lesion site can be observed after stroke onset. Such neuroinflammation within the brain parenchyma represents the innate immune response, as well as the result of the additional interactions between peripheral and resident immune cells. Accumulative studies have illustrated that the pathological process of ischemic stroke is associated with resident and peripheral immunity. The infiltration of peripheral immune cells within the brain parenchyma implicitly contributes to secondary brain injuries. Therefore, better understanding of the roles of resident and peripheral immune reactions toward ischemic insult is necessary. In this review, we summarized the interaction between peripheral and resident immunity on systemic immunity and the clinical outcomes after stroke onset and also discussed various potential immunotherapeutic strategies.

## 1 Introduction

Stroke is a disease with cerebral blood flow obstruction (ischemic stroke) or cerebral vascular rupture hemorrhage (hemorrhagic stroke) caused by a variety of factors (1). Among which, ischemic stroke accounts for approximately 87% and is the leading cause of mortality and morbidity (2). According to data from the World Health Organization (WHO), approximately 5 million people die of ischemic stroke worldwide each year, and about 1 million people lose their lives due to ischemic stroke in China each year (3, 4). Although tremendous efforts have been made with regard to therapeutic strategies, a number of treatments have not been successfully translated into clinical settings (5). This disappointing outcome indicates an insufficient understanding of the pathophysiology of ischemic stroke and emphasizes the need to determine the underlying mechanisms of the progression of brain injuries (6).

Numerous studies have revealed the activation of the innate and adaptive immune responses in the brain lesion of ischemic stroke. The stroke onset induces sustained immune reactions during both the acute and chronic phases. Accumulative evidence has revealed that neuroinflammation plays the predominant role in the progression of brain injury (7). A variety of harmful substances, including excessive cytokines/chemokines and reactive oxygen species (ROS), comprise the stroke-induced inflammatory cascade, which are responsible for the damaged vascular integrity, cell death, and secondary brain damage (8). However, rapid and optimal neuroinflammation is also indispensable in the subsequent process of injury repair and functional recovery (9). Such dual characteristics may be due to systemic conditions and the time-dependent role of immune reactions (9). Furthermore, regional and systemic immune responses present spatial and temporal functions during the different phases after stroke. In this review, we discuss the interaction between the peripheral and resident immune cells and summarize the immunotherapeutic strategies against ischemic stroke to better understand the role of immune reaction in the progression of stroke.

## 2 Resident and peripheral immune responses in ischemic stroke

The immune reactions within the brain parenchyma are distinguished from periphery immunity. In the past decades, accumulative evidence has revealed the link of immune responses between the brain and the periphery. With the physical defense of the blood–brain barrier (BBB), lymphatic drainage and antigen-presenting cells (APCs) are deficient, which makes the brain an immune-privileged organ (10–12). However, after the disrupture of the BBB, peripheral immune cells can move into the lesion sites. Cellular or soluble components within the central nervous system (CNS) are drained through the cerebrospinal fluid (CSF) into the deep cervical lymph node and induce further immune reactions. Such neuroinflammatory interaction will be intensified in a variety of neurological conditions, especially in stroke. It has been reported that peripheral immune cells can also be observed in the brain parenchyma after stroke onset (13). Therefore, it is necessary to fully examine the physiological interactions between the immune responses and brain injuries.

**Figure 1:**
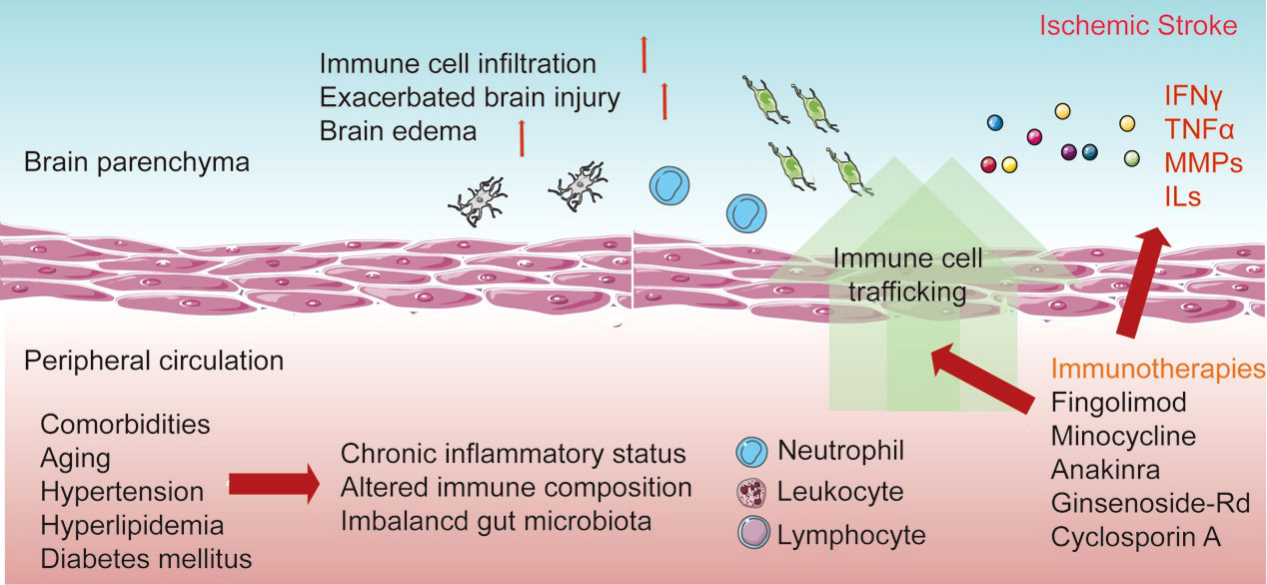
Interaction between resident and peripheral immune cells in response to stroke. The distinction between resident and peripheral immunity is lost after the disruption of the blood–brain barrier (BBB). The resident microglia is then activated after recognition of the danger signals in the lesion sites and the release of pro-inflammatory cytokines. Additionally, the increase of adhesion molecules enables the migration and invasion of peripheral immune cells. Invading neutrophils, monocytes, and lymphocytes elicit neuroinflammation and influence the development of brain injury and the recovery process. Additionally, comorbidities occur with the chronic inflammatory status in peripheral circulation. Currently, various immune-based therapies have been established to improve the acute damage and recovery process in ischemic stroke.

After stroke occurrence, harmful substances and peripheral antigens enter the brain parenchyma through the disrupted BBB, followed by the subsequent recruitment of immune cells into the lesion sites, resulting in activated neuroinflammation. Due to the influence of immune and neuroinflammatory reactions on the progression of injuries, mortality, and recovery from stroke, a better understanding of the compartmentalization and the links between the brain and peripheral immune reactions is necessary, which will facilitate the establishment of effective therapeutic strategies through immune-targeted approaches (**Figure 1**).

### 2.1 Activated immune reactions in the brain after ischemic stroke

According to a previous study, the infiltration and the activation of immune cells caused by ischemic stroke are temporally and spatially regulated (14). The biological function of these immune cells depends on the microenvironment during the different phases after stroke onset. At the earliest phase, within hours of ischemic onset, the resident microglia and astrocytes are both remarkably activated, which is also maintained in the following weeks. In a previous longitudinal transcriptome analysis, the sustained innate and adaptive immune transcripts in the brain of patients with ischemic stroke were determined (15). A variety of harmful substances and excessive cytokines will further lead to a wide range of cell death. Of note is that the damage-associated molecular patterns (DAMPs) derived from damaged cells can be recognized by pattern recognition receptors (PRRs), which in turn activate further innate inflammatory reactions (16). Such activated immune cells accompanied by damaged cells release various inflammatory substances, which leads to the entry and infiltration of peripheral immune cells. In addition, the infiltration of peripheral immune cells can be observed at the acute, subacute, and recovery phases of ischemic stroke, indicating long-term immune reactions. However, the spatial and temporal patterns of immune trafficking during the recovery period are still not fully defined. Activation of the NLRP3 inflammasome is also strongly associated with immunotherapy after ischemic stroke. NLRP3 has been thought to be a key factor in neuronal injury after stroke. NLRP3 inflammasomes can produce large amounts of inflammatory factors after ischemic stroke, ultimately leading to neurological dysfunction and neuronal cell death (17). Targeting the upstream and downstream NLRP3 pathways has shown promise in the treatment of ischemic stroke (18).

### 2.2 Innate and adaptive immunity after ischemic stroke

Innate immunity represents the initial immune reaction to ischemic stroke onset, while peripheral immune cells can be observed within the brain parenchyma in the later phase (19, 20). According to a previous study based on experimental ischemic stroke models, peripheral immune cells can be detected in the lesion site after stroke and last for at least 7 days (21). Of these peripheral immune cells, neutrophils are the first invading cells into the brain parenchyma. Subsequently, monocyte-derived macrophages (MDMs), dendritic cells, and some other immune cells are found in the damaged sites after ischemic stroke (22). In the later stages, adaptive immunity will be activated, which features the infiltration of T and B lymphocytes within the brain parenchyma (23). Accumulative evidence has revealed the roles of the innate and adaptive immunity in the progression of stroke, even affecting the recovery stage. In this section, we will summarize the role of various immune cells after stroke onset.

#### 2.2.1 Infiltrated neutrophils

Neutrophils are the first immune cells into the lesion site after ischemic stroke onset, which can be observed at 30 min and peak at 24–72 h post-stroke, decreasing over time (24, 25). The infiltration of neutrophils is considered a risk factor for poor outcomes, which accounts for the release of various mediators including metalloproteases (MMPs), ROS, and the pro-inflammatory interleukin-1 beta (IL-1β). Although the relationship between neutrophils and ischemic lesions is well recognized based on clinical trials and experimental studies, conflicting roles of neutrophils are still revealed in ischemic outcomes (26). There are two classical subtypes of neutrophils—neurotoxic phenotype and neuroprotective phenotype—the different polarized status of which is based on functional heterogeneity (27). Moreover, a variety of substances, including ROS, are produced, further inducing the release of pro-inflammatory cytokines and activating neuroinflammation (28). Additionally, the anti-inflammatory effects of neutrophils have also been reported through the degradation of pro-inflammatory substances, which is beneficial to brain recovery post-ischemic stroke. Neutrophils that present signs of alternative activation, which features the expressions of Arg1 and YM1, might be beneficial in the recovery from stroke. Cytotoxic neutrophils are generated through the activation of the TLR4 signal pathway, while myeloid-selective TLR4 knockout promotes the polarization of cytoprotective neutrophils and plays a neuroprotective function (29). Due to the functional heterogeneity of neutrophils, neutrophil manipulation may be a potential therapeutic strategy for patients with stroke.

#### 2.2.2 Microglia and monocyte-derived macrophages

The microglia and MDMs are the dominant immune cells that produce and release a wide range of cytokines or chemokines with a peak at 3–7 days after stroke (30, 31). They play an essential role as the predominant innate immunity in the regulation of neuroinflammation after ischemic stroke onset. However, according to experimental studies, the microglia and MDMs also play a detrimental role in the progression of neuronal death (31). It is well known that the microglia and MDMs can release ROS and various inflammatory substances, which are cytotoxic and induce the progression of brain injury (32, 33). Moreover, enhanced phagocytosis of cells is closely related to brain damage after ischemic stroke. However, the removal of immunogenic intracellular contents and apoptotic cells can inhibit cytotoxicity and attenuate the progression of brain damage. Additionally, resident phagocytes within the brain parenchyma can take up infiltrated neutrophils, which have been revealed to be beneficial in the recovery from brain injury post-stroke. There is also conflicting evidence on the neuroprotective function of the microglia/macrophages after stroke. It has been demonstrated that, after depletion or inactivation of the microglia or MDMs, tissue repair will be inhibited, but the neuroinflammation and secondary brain injury will be accelerated, accounting for the presence of a variety of anti-inflammatory cytokines such as IL-10 and TGF-β (produced and secreted by the microglia and MDMs), which are responsible for tissue recovery (34). Additionally, several common biomarkers are localized in the microglia, which indicates the overlapping function of neuroinflammation and immune regulation. The dual function of mononuclear phagocytes depends on bidirectional polarization. Within the lesion site, the microglia and MDMs can be activated and polarized toward either the pro-inflammatory M1-like phenotype (classically activated subtype) or the anti-inflammatory M2-like phenotype (alternatively activated subtype) (35). The M1 phenotype comprises the pro-inflammatory immune cells that are responsible for ischemic damage and poor clinical outcomes, while the M2 phenotype contributes to the recovery after stroke through the release of various anti-inflammatory substances. The microglia can be polarized into different states within a few hours after stroke onset (36). In acute ischemia–reperfusion assays, the microglia is polarized from the M1 to the M2 phenotype by drugs that inhibit the secretion of pro-inflammatory cytokines and promote the expression of anti-inflammatory cytokines (36, 37). The M1 phenotype is pro-inflammatory and secretes IL-6, tumor necrosis factor (TNF), and IL-1β, which contribute to brain injury after stroke (38, 39). The M2 phenotype has anti-inflammatory effects and secretes anti-inflammatory cytokines such as IL-10, IL-4, and transforming growth factor beta (TGF-β), which further protect neurological function and improve prognosis after stroke (38, 40). The peak time points of cytokine expression differ in different cell polarization states. Promotion of the microglia M2 polarization is one method for exerting neuroprotective effects in ischemic stroke (41).

#### 2.2.3 NK cells

Natural killer (NK) cells are important members of the innate immunity. NK cells can immediately respond to pathological insults after stroke onset without a prior activation period, which enhances neuroinflammation and further exacerbates brain injury. The expression of CXC3CR1 on NK cells is required for the recruitment of neutrophils, which is also dependent on the expression of interferon gamma (IFN-γ) (42). Moreover, according to a previous study, IFN-γ can be secreted from NK cells and recruit macrophages or dendritic cells, which are involved in secondary ischemic damage (13). However, there are also several conflicting reports on the biological role of NK cells in the progression of stroke. In 2014, Mracsko et al. demonstrated that the depletion of NK cells presented no beneficial effects after ischemic stroke in a rodent middle cerebral artery occlusion (MCAO) model (43). Furthermore, the administration of IFN-γ prolongs survival after stroke with the function of antibacterial infection rather than pro-inflammation (44).

#### 2.2.4 T and B lymphocytes

Immediately after stroke onset, brain-derived antigens are generated from damaged cells and reach peripheral circulation. Subsequently, T and B lymphocytes will be stimulated in the spleen and lymph nodes. Compared with that of other immune cells, the infiltration rate of T lymphocytes into the lesion sites is relatively lower. Dead cells are taken up by phagocytes and present antigens, which induce the migration of T lymphocytes into the ischemic region within a few days after stroke (45). T lymphocytes have been demonstrated to show detrimental effects after stroke, while their depletion played a protective role in a rodent ischemic stroke model (46). Regulatory T cells (Tregs) are another predominant member of T lymphocytes that are also protective effectors in the progression of ischemic stroke. Tregs play a neuroprotective role after stroke onset through the release of various anti-inflammatory substances and through maintaining the BBB integrity (47). Tregs are locally expanded and are dependent on the activation of serotonin signaling. Their main role is the maintenance of immune homeostasis and the attenuation of the onset of overpowering immune responses (48). It has been shown that the number of Tregs in normal brain tissue is relatively low, whereas after ischemic stroke, a large number of Tregs accumulate in the brain and play a neuroprotective role until the chronic phase of stroke, which is essential for neurological recovery from ischemic stroke (49, 50). Tregs mainly infiltrate the brain 1–5 weeks after stroke and remain at high levels for about a month (51). In the acute phase, within 1 week after stroke, Tregs may reduce the inflammatory activation through the production of the anti-inflammatory cytokine IL-10 (52). The depletion of Tregs within 1 week after stroke can inhibit neural stem cell proliferation. During the chronic phase of stroke (after 1 week), a significant reduction in the number of Tregs was seen in mice treated with an inhibitor of T cells, and eventual neurological recovery was delayed (53). Therefore, increasing the number of Tregs is likely to be a reliable method to improving the neurological function in the acute and chronic phases after stroke.

Accumulative studies have illustrated the role of T and B lymphocytes in the progression of secondary brain injury. It has been reported that CD4^+^ or CD8^+^ T lymphocytes and B cells increase within 4 days after stroke (54). Additionally, the depletion of CD4^+^ or CD8^+^ T lymphocytes with monoclonal antibodies also alleviates the progression of brain damage after stroke onset (55). A variety of studies demonstrated that T lymphocytes clonally expand within the brain parenchyma or peripheral circulation in the first week after stroke based on antigen-dependent activation (55). However, another study indicated that T lymphocytes acerbate the ischemic damage without the involvement of antigen recognition or co-stimulatory pathways (56). In the acute phase of stroke, Tregs also play a critical role in the inhibition of neuroinflammation and the other pro-inflammatory T-lymphocyte subpopulations (57). Interestingly, a previous study also reported the detrimental role of Tregs in ischemic stroke. Targeting the depletion of Tregs attenuated the ischemic damage and improved the neurological function, while microvascular dysfunction was exacerbated (58). B-lymphocytic responses can occur in the late stage of ischemic stroke. The production of CNS antibodies increases over time, and antibody synthesis can be observed within the first week in approximately half of stroke survivors (59).

## 3 Comorbidity and immunity after stroke onset

A wide range of comorbidities involving aging, hypertension, hyperglycemia, and hyperlipidemia are linked to the increased incidence of cardio- and cerebrovascular diseases (60). There is consistent accumulative evidence that such comorbid conditions can impair the immune reactions in peripheral circulation. Various cascades, including oxidative stress, edema, and lipid oxidation, are involved in the pathological progression of stroke, which are also converged into immune responses (8). In comorbid conditions, the inflammatory reactions will be intensified and will negatively affect the clinical outcomes of ischemic stroke (61–63).

Aging compromises the immune responses after brain damage. In particular, both clinical and preclinical studies have reported that aging diminishes the phagocytic function of monocytes, reduces the chemotaxis of neutrophils, and inhibits the cytotoxic role of NK cells (64–66). In terms of adaptive immunity, the number and the function of lymphocytes are also altered with aging (67). Hypertension is the second common risk factor for stroke that is associated with poor outcomes and higher mortality (68, 69). It has been illustrated that hypertension-related stroke is associated with the involved autoregulatory responses, the dysfunction of vessels, and enhanced oxidative stress (70, 71). Patients with hypertension present exacerbated clinical outcomes after stroke due to the deregulation of chronic systemic inflammation. It has been reported that hypertension can activate monocytes and elevate the production of ROS (72). Increased CD45^+^ cells and worse neurological injuries can be observed in hypertension after stroke onset (73). Moreover, hypertension can also activate the microglia, endothelial cells, and astrocytes within the brain parenchyma. Additionally, it has been reported that high levels of cholesterol in plasma are linked to the higher occurrence of vascular diseases and to poor clinical outcomes after stroke (74). Similar to other comorbidities, the negative influence of hyperlipidemia after stroke injuries is related to the chronic inflammatory environment in both the periphery and brain parenchyma. Accumulative evidence has demonstrated that high levels of cholesterols in plasma are positively associated with a larger infarction area and a higher rate of edema formation (75). The hyperlipidemia-induced damage is closely associated with the elevated pro-inflammatory reactions within the lesion sites, which are recognized by the multifunctional class B scavenger receptor, CD36, after stroke (76). In addition, clinical trials have also revealed that hyperlipidemia is related to worse neurological outcomes of patients with stroke, while the administration of cholesterol-containing drugs can attenuate such poor outcomes due to their pleiotropic effects on vascular integrity, neuroinflammation, and oxidative stress (77). Lastly, diabetes mellitus (DM) is also a high risk factor for patients with stroke, which features hyperglycemia and insulin resistance, as well as chronic systemic inflammation (78). Accumulative evidence has illustrated the effects of DM on the systemic pro-inflammatory status and the alteration of the immune reactions after stroke. Additionally, it has also been demonstrated that diabetes can serve as a predictor for the poor clinical outcomes of patients with ischemic stroke, which can indicate a larger infarct volume, the occurrence of brain edema, and damaged neurological function.

## 4 Immune-based strategies against ischemic stroke

Accordingly, the immune responses are closely associated with tissue recovery and clinical outcomes after stroke. With the disruption of the BBB, the distinction between resident and peripheral immune cells is also lost, which provides a unique opportunity to modulate the pathological or recovery process. In this section, we provide insights into the several potential drugs in clinical trials that work by targeting the immune system (**Table 1**).

**Table 1:** Potential clinical immunotherapeutic strategies *NIHSS*, National Institutes of Health Stroke Scale; *mRs*, modified Rankin scale.

Candidate drug	Trial type	Dosage	Main evaluation criteria	Outcomes	Year	Reference
Fingolimod	Prospective, multicenter, randomized, open-label, blinded endpoint clinical trial	0.5 mg daily for 3 consecutive days	NIHSS score at 24 h	Better early clinical improvement at 24 h	2018	Tian et al. (79)
Minocycline	Open-label, evaluator-blinded study	200 mg/day	Baseline to day 90 in NIHSS	A significantly better outcome with minocycline treatment compared with placebo	2007	Lampl et al. (80)
Anakinra	Randomized, double-blind, placebo-controlled trial	2 mg kg^−1^ h^−1^ infusion over 72 h	NIHSS score and mRs score	Markers of biological activity were lower in the pilot group.	2005	Emsley et al. (81)
Ginsenoside-Rd	Randomized, double-blind, placebo-controlled, phase II multicenter trial	10 and 20 mg/day for 14 consecutive days	NIHSS score at 15 days	NIHSS scores were significantly different in the pilot group.	2009	Liu et al. (82)
Cyclosporin A	Multicenter, single-blinded controlled trial	2.0 mg/kg for 30 days	Infarct volume on MRI at 30 days	Smaller infarct volume could be observed in some patients.	2015	Nighoghossian et al. (83)

### 4.1 Fingolimod

Inflammation is closely related to the pathogenesis of ischemic stroke, and the recruitment of inflammatory cells can further aggravate brain damage (84). Fingolimod is one of the sphingosine 1-phosphate receptor (S1PR) modulators and is the first S1PR modulator approved for the treatment of multiple sclerosis (85). In the past few years, fingolimod has gradually been found to be a potentially beneficial drug in the treatment of stroke. According to previous studies, fingolimod can limit the migration and circulation of lymphocytes (86). In some clinical trials, fingolimod showed beneficial effects in ischemic stroke. In the study of Tian et al., fingolimod was found to enhance the effect of alteplase administration in the short-acting time window (4.5–6 h) primarily by promoting anterograde reperfusion and retrograde collateral flow (79). Fingolimod primarily maintains the integrity and function of microvessels by inhibiting the migration of lymphocytes into the brain parenchyma, further reducing the occurrence of vascular inflammation and inhibiting the formation of inflammatory thrombus in capillaries, finally maintaining the perfusion of brain tissue and rescuing the post-stroke penumbra (87, 88). In another early-phase clinical study, participants given fingolimod showed lower circulating lymphocyte counts, milder neurological deficits, and better recovery of neurological functions (89). In addition, patients using fingolimod did not show obvious drug side effects, indicating the safety of the drug. Furthermore, fingolimod can reduce peri-hematoma edema after hemorrhagic stroke and can further improve the clinical prognosis of patients (90). However, in some other studies, fingolimod was not found to confer long-term treatment benefits in an acute intracerebral hemorrhage (ICH) mouse model, and its effect on ICH was weaker than that of ischemic stroke (91). A number of large medical institutions are still conducting clinical trials of fingolimod for the endovascular treatment of ischemic stroke. In summary, some of the latest research studies have shown the role of fingolimod in stroke to be very promising. The satisfactory effect of fingolimod in ischemic stroke is greatly anticipated.

### 4.2 Minocycline

Minocycline is a broad-spectrum antibacterial tetracycline antibiotic that achieves an antibacterial effect by combining with transfer RNA (tRNA) (92). The lipophilic nature of minocycline allows it to cross the BBB and exert its effects on the brain (93). Some previous studies have shown that minocycline can confer neuroprotective effects in a variety of neurological diseases, including ischemic stroke (94–96). Previous preclinical trials have fully demonstrated that minocycline can improve the prognosis of patients with acute ischemic stroke. In rat models of stroke, minocycline treatment protected the brain by affecting the glial cells surrounding the injured brain (97). Astrocytes and the microglia have great influence on the plasticity of neurons around infarction. Cell death after infarction can release pro-inflammatory factors and affect scar formation by activating peripheral glial cells (98, 99). Minocycline can reduce the infarct size and improve neurological function when administered in the acute phase of stroke, and it can also be used in combination with a tissue plasminogen activator (100). Some clinical trials have also confirmed the neuroprotective effect of minocycline in ischemic stroke. The combination of minocycline with other treatment modalities increased the success rate of stroke treatment. In a meta-analysis of randomized controlled clinical trials (including seven randomized controlled trials), minocycline showed efficacy in patients with acute stroke and appeared to be a promising neuroprotective agent (101). In a large open-label, evaluator-blinded study, patients who received minocycline showed significantly better post-stroke assessments compared with the placebo group (80).

### 4.3 Anakinra

Anakinra is an IL-1 receptor antagonist. Previous studies have fully described IL-1 to play an important role in the development of brain damage in ischemic stroke (102). As the main members of the IL-1 family, IL-1α and IL-1β play an important role in stroke. A previous study confirmed that genetic deletion of IL-1α and IL-1β in mammals leads to a substantial reduction in damage after experimental stroke (102). The pro-inflammatory cytokine IL-1 promotes destructive inflammation in brain regions after stroke (103). In addition, due to the damage of the BBB after stroke, white blood cells and inflammation-related factors can more easily enter the brain through the BBB. The interleukin-1 receptor antagonist (IL-1Ra), which inhibits IL-1, is neuroprotective in stroke models (104). In a randomized, double-blind, placebo-controlled trial, peripheral administration of anakinra 6 h after the onset of acute stroke significantly reduced neuronal cell death and the inflammatory processes, demonstrating its therapeutic potential and its safety and efficacy in acute stroke (81).

### 4.4 Ginsenoside-Rd

Ginsenoside-Rd is derived from the well-known traditional Chinese medicine ginseng (105). A lot of previous studies, including some clinical trials, have confirmed its beneficial effects on stroke. Ginsenoside-Rd can alleviate brain damage after stroke by inhibiting inflammation (106). Ginsenoside-Rd also has the effect of penetrating the BBB, further exerting effects on brain tissue (107). In experimental models of stroke (mainly transient middle cerebral artery occlusion, tMCAO), treatment with ginsenoside-Rd before or after ischemic stroke reduced the cerebral infarct volume, increased the survival ratio of functional neurons, and protected neurological function (108–110). A treatment trial with ginsenoside-Rd (10 mg/kg) found that it could inhibit poly(ADP-ribose) polymerase-1, thereby downregulating the apoptosis-inducing nuclear factor-kappa B p65 subunit nuclear accumulation in MCAO mice, which supports the anti-inflammatory therapeutic effect of ginsenoside-Rd in ischemic stroke (111). It has also been shown to reduce neuroinflammation after stroke by downregulating the activation of the microglia (108). In a phase II randomized, double-blind, placebo-controlled multicenter trial, patients with stroke were divided into a placebo group, low-dose group (10 mg), and a high-dose group (20 mg), and the 15-day National Institutes of Health Stroke Scale (NIHSS) score was used as the primary endpoint. The results showed significant statistical differences between the ginsenoside-Rd group and the control group, which indicated that ginsenoside-Rd may play a neuroprotective role in acute ischemic stroke (82). In a subsequent phase III trial, the primary endpoint set was the distribution of the disability scores on the modified Rankin scale (mRs) at 90 days, and the results also indicated the same conclusion: that ginsenoside-Rd can improve the prognosis of patients with acute ischemic stroke (112).

### 4.5 Cyclosporin A

Cyclosporin A is generally used as an immunosuppressant, and it is primarily used clinically for the treatment of autoimmune diseases and organ transplant rejection. Moreover, cyclosporin A can also act on neural precursor cells in neurogenic regions of the brain (113, 114). Recent studies have found that cyclosporin A can act as a neuroprotective agent in stroke models by activating neural precursor cells (114). It can also inhibit mitochondrial dysfunction caused by ROS formation after stroke (115). In addition, treatment with chronic cyclosporin A has shown positive effects on cognitive recovery after stroke. The earliest research on the anti-ischemic effect of cyclosporin A in a MCAO animal model was conducted in the 1990s, with the experimental results showing that the oral administration of cyclosporin A in animal models of cerebral ischemia could significantly reduce the volume of cerebral infarction and edema (116). The role of cyclosporin A has also been confirmed in subsequent clinical studies. In a multicenter, single-blind controlled trial, patients with stroke received intravenous injection of cyclosporin at 2.0 mg/kg, and the results showed that, although cyclosporine was not effective in reducing the infarct volume, a smaller infarct volume and effective recanalization were observed in some patients with proximal cerebral artery occlusion (83). Due to its basic foundation and clinical research prospects, there is a high expectation for cyclosporin A in the treatment of ischemic stroke.

## 5 Conclusion and perspective

Excessive activation of immune reactions may lead to harmful neuroinflammation, while insufficient immunity will result in infection. Immunity maintains balance under healthy conditions; however, following several pathological events, especially stroke, such balance may be disrupted. The inflammatory reactions after stroke are closely related to secondary brain injuries and poor clinical outcomes. Accordingly, great effort has been made to alleviate excessive neuroinflammation within the brain parenchyma; however, the relevance of systemic immunity has not been addressed. The distinction between the resident immune cells within the brain parenchyma and systemic immunity is lost; consequently, the neuroinflammation will interact with peripheral immunity. The infiltration of peripheral immune cells plays an indispensable role after ischemic insult, and the neuroinflammation within the damaged brain inevitably regulates the systemic immunity. So far, the primary studies have solely focused on the local neuroinflammation after stroke and have neglected the systemic immunity and the interaction between organs. Although anti-inflammatory therapeutic strategies can effectively alleviate neuroinflammation, the other effects on systemic immunity also need to be considered in future explorations.

## Author contributions

WC designed the study and drafted the manuscript. QZ produced the figure and table. CL and YX revised the manuscript. All authors contributed to the article and approved the submitted version.
